# Severe upper gastrointestinal involvement in ulcerative colitis successfully treated with sequential therapy using intravenous tacrolimus and upadacitinib: a case report

**DOI:** 10.3389/fmed.2026.1812628

**Published:** 2026-05-08

**Authors:** Kenji Kinoshita, Keisei Koishi, Masaki Nagaya, Masayuki Higashino, Sonoe Yoshida, Takahiro Yamamura, Kosuke Nagai, Kazuteru Hatanaka, Yoshiya Yamamoto, Takehiko Katsurada, Naoya Sakamoto

**Affiliations:** 1 Department of Gastroenterology, Hakodate Municipal Hospital, Hakodate, Japan; 2 Department of Gastroenterology and Hepatology, Hokkaido University Graduate School of Medicine, Sapporo, Japan

**Keywords:** sequential therapy, tacrolimus, ulcerative colitis, upadacitinib, upper gastrointestinal involvement

## Abstract

Ulcerative colitis (UC) is classically regarded as a disease limited to the colon; however, upper gastrointestinal (UGI) involvement has been increasingly recognized. We report a rare and clinically severe case of UC with marked UGI involvement that was successfully treated with tacrolimus and subsequently maintained with upadacitinib. A 19-year-old man presented with epigastric pain, diarrhea, and elevated inflammatory markers. Computed tomography demonstrated diffuse gastric wall thickening, whereas colonic involvement was relatively mild. Esophagogastroduodenoscopy revealed extensive erythema, spontaneous bleeding, and ulcerations in the stomach and duodenum, whereas ileocolonoscopy showed findings consistent with moderate UC. Histopathological examination of gastric and duodenal biopsies demonstrated dense inflammatory cell infiltration with crypt abscesses, supporting a diagnosis of UC-associated UGI involvement. The patient was refractory to high-dose corticosteroids and unable to tolerate oral therapy. Continuous intravenous tacrolimus was initiated, resulting in clinical and endoscopic improvement. Due to tacrolimus-related adverse effects, maintenance therapy was transitioned to upadacitinib, leading to sustained clinical and endoscopic remission. This case highlights the importance of recognizing severe UGI involvement as a rare UC phenotype and suggests that aggressive immunomodulatory therapy, including calcineurin inhibitors and Janus kinase inhibitors, may be effective in selected patients.

## Introduction

Ulcerative colitis (UC) is a chronic inflammatory bowel disease (IBD) primarily affecting the colon and rectum, characterized by alternating periods of remission and relapse (1). Recently, several studies have reported upper gastrointestinal (UGI) involvement in UC (2–11); however, there is currently no established consensus regarding the diagnosis or management of UC-associated UGI lesions. Here, we present a rare and clinically severe case of UC with marked UGI involvement, in which the patient achieved both clinical and endoscopic remission using advanced therapies, including calcineurin inhibitors and Janus kinase inhibitors. This study was approved by the Institutional Review Board of Hakodate Municipal Hospital (IRB No. 2025-299) and was conducted in accordance with the principles of the Declaration of Helsinki. The requirement for written informed consent was waived because of the retrospective nature of this case report.

## Case presentation

**FIGURE 1:**
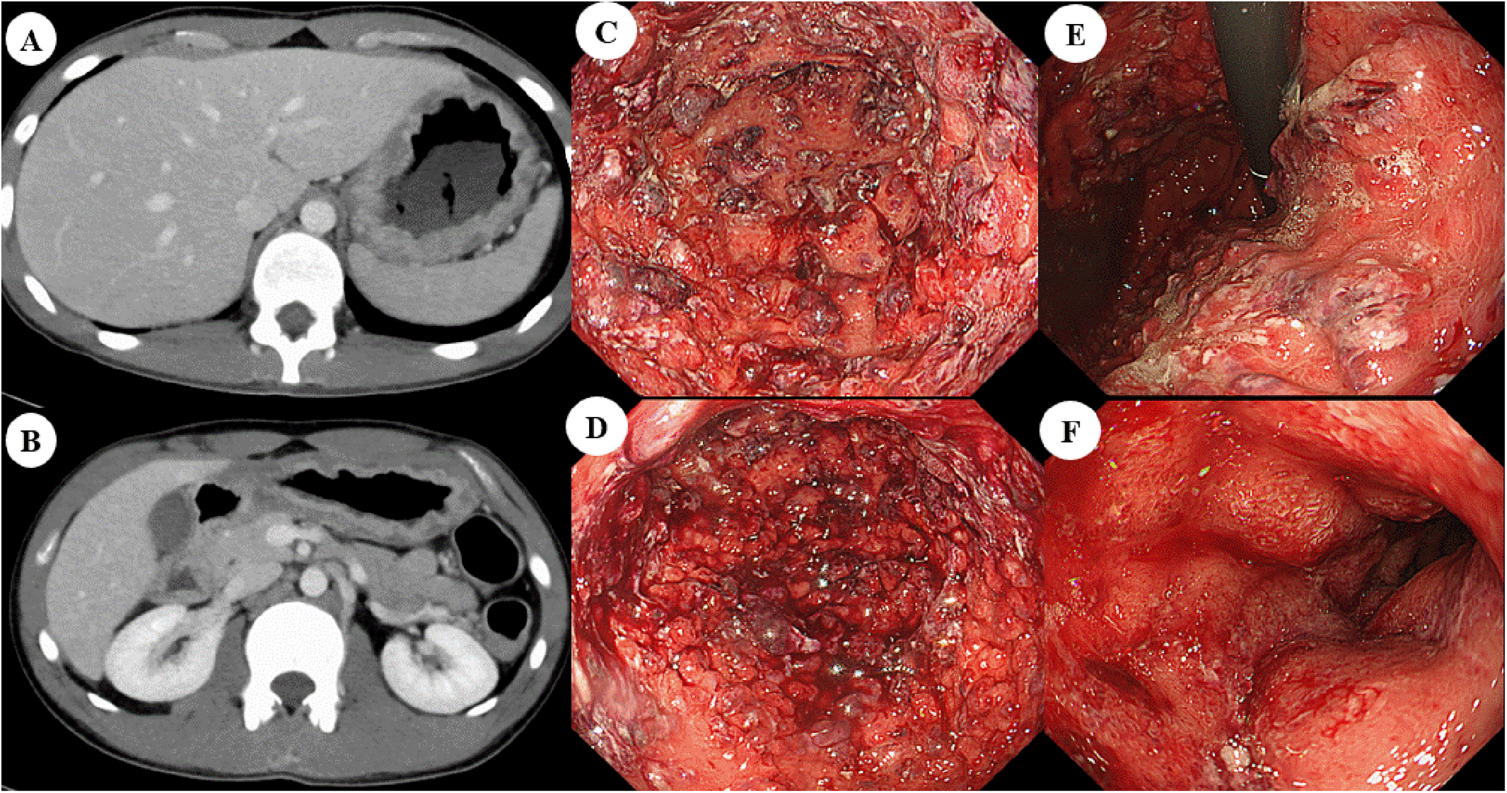
Radiologic and endoscopic findings of severe upper gastrointestinal involvement at initial presentation. **(A,B)** Contrast-enhanced computed tomography demonstrating diffuse gastric wall thickening extending from the fornix to the antrum. **(C–F)** Esophagogastroduodenoscopy showing extensive erythema, spontaneous bleeding, and irregular ulcerations in the upper gastrointestinal tract: **(C)** gastric antrum, **(D)** gastric body, **(E)** lesser curvature of the stomach, and **(F)** duodenal bulb.

**FIGURE 2:**
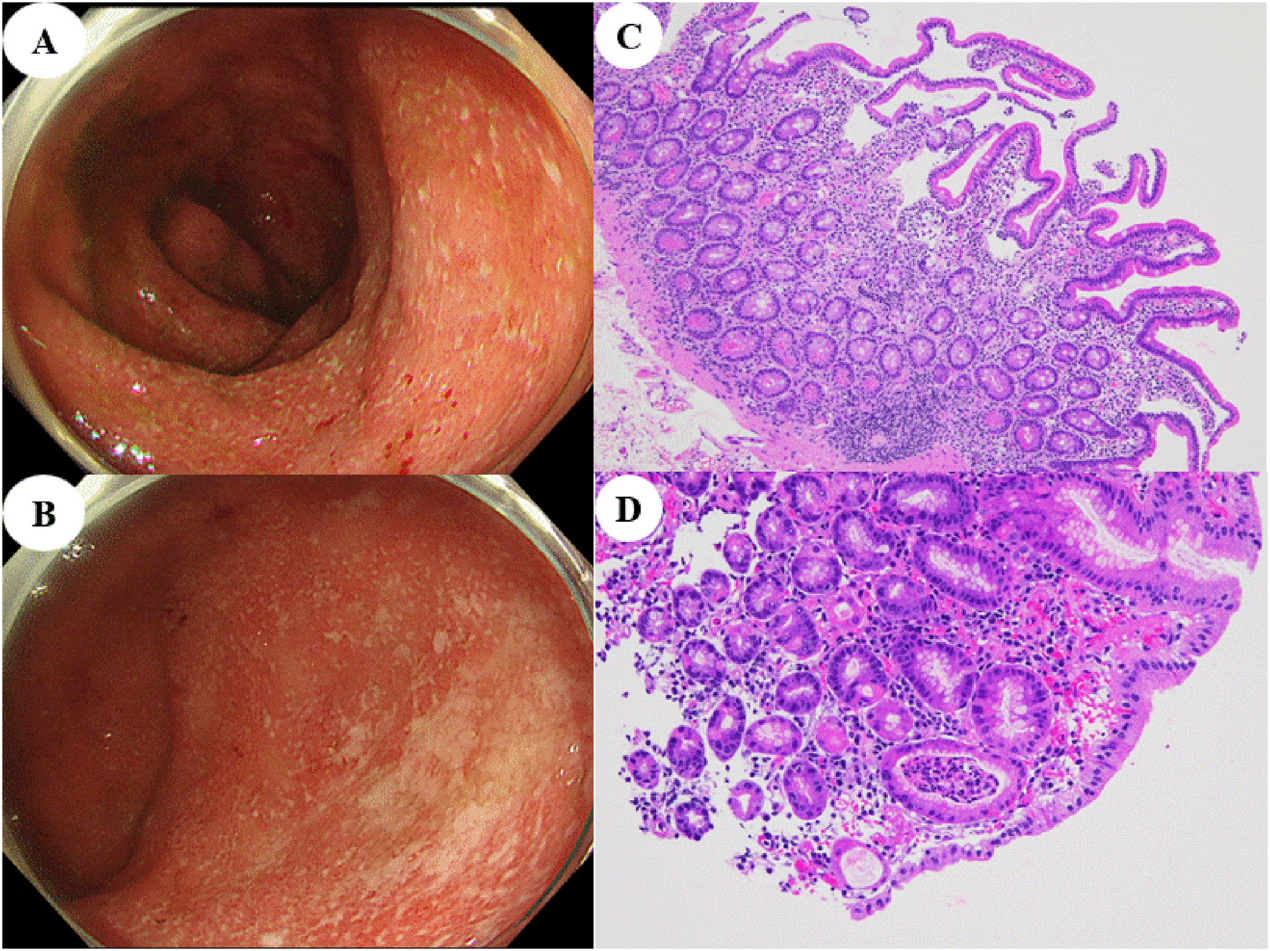
Ileocolonoscopic and histologic findings at presentation. **(A,B)** Ileocolonoscopic findings showing diffuse erythema and loss of vascular pattern, consistent with moderate ulcerative colitis: **(A)** ascending colon and **(B)** rectum. **(C,D)** Histologic findings of gastric biopsies demonstrating active inflammation: **(C)** gastric mucosa with dense inflammatory cell infiltration [hematoxylin and eosin (H&E) staining, ×10] and **(D)** higher magnification revealing crypt abscess formation (H&E staining, ×20).

A 19-year-old man with no significant past medical history presented with a 1-month history of persistent epigastric pain and diarrhea. Despite receiving symptomatic therapy at a local clinic, his condition did not improve, and he was transferred to our hospital on an emergency basis. On admission, the patient presented with severe epigastric pain, along with frequent diarrhea and bloody stools. The Lichtiger index was 10, indicating moderate to severe disease activity. Laboratory analysis revealed elevated inflammatory markers, including a white blood cell count of 20,200 /μL and a C-reactive protein (CRP) level of 11.0 mg/dL. The hemoglobin level was 14.1 g/dL, and the serum albumin level was 2.7 g/dL. Computed tomography (CT) showed mild thickening of the entire colonic wall as well as pronounced thickening of the gastric wall (Figures 1A, B). Esophagogastroduodenoscopy (EGD) revealed prominent erythema in both the stomach and duodenum, accompanied by spontaneous bleeding and partial ulceration (Figures 1C–F). Ileocolonoscopy showed diffuse colonic erythema, purulent exudates, and multiple erosions, findings consistent with moderate UC (Figures 2A,B). Despite only moderate colonic disease activity, the upper gastrointestinal lesions were extensive and unusually severe. Histopathological examination of biopsy specimens from colonic lesions showed diffuse neutrophilic infiltration with cryptitis-like changes and architectural distortion. Duodenal and gastric biopsy specimens revealed dense inflammatory cell infiltration consisting of lymphocytes, plasma cells, neutrophils, and eosinophils. In the gastric fundic gland mucosa, marked active inflammation with edema was observed, and crypt abscesses were occasionally identified (Figures 2C,D). Deeper sections showed no evidence of granuloma formation, cytomegalovirus inclusion bodies, Helicobacter pylori organisms, or malignant findings. Based on these findings, a diagnosis of UC with severe UGI involvement was established.

**FIGURE 3:**
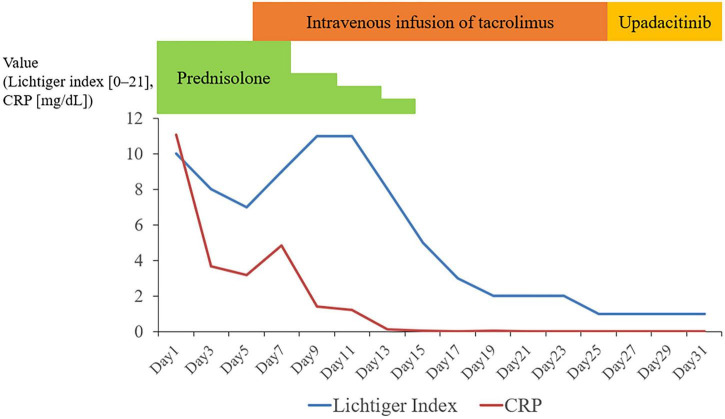
Clinical course and treatment timeline. Temporal changes in the Lichtiger index and serum C-reactive protein (CRP) levels in relation to treatment. High-dose corticosteroids failed to induce remission, whereas continuous intravenous tacrolimus resulted in rapid clinical and biochemical improvement. The patient was subsequently transitioned to upadacitinib due to adverse events, leading to sustained remission. CRP, C-reactive protein.

Initial treatment with prednisolone (1 mg/kg) failed to elicit an adequate clinical response, indicating a steroid-refractory state. Although oral tacrolimus was considered, severe nausea and epigastric pain rendered oral administration unfeasible. Therefore, on day 6 of hospitalization, continuous intravenous infusion of tacrolimus was initiated at a dose of 0.02 mg/kg/day, with subsequent adjustments made to maintain a tacrolimus trough level of approximately 20 ng/mL. Blood tacrolimus trough levels were monitored daily during the induction phase, and laboratory parameters, including renal function and electrolytes, were assessed every 1–2 days to ensure safety and appropriate dose adjustment. After 3 weeks, endoscopic evaluation revealed significant improvement in both UGI and colonic lesions. However, the patient developed mild renal dysfunction and hypomagnesemia during tacrolimus therapy, prompting a transition to upadacitinib. Serum creatinine increased to 1.4 mg/dL (baseline: 0.8 mg/dL), and serum magnesium decreased to 1.1 mg/dL. These abnormalities were mild and improved following discontinuation of tacrolimus with supportive management, including intravenous fluid administration and electrolyte replacement. Upadacitinib was initiated at 45 mg once daily. Although clinical remission was achieved within 2 months, the induction dose was continued for a total of 4 months due to the severity of disease and concern for early relapse, followed by dose reduction to 30 mg for maintenance therapy. The patient subsequently achieved sustained clinical remission (Figure 3).

**FIGURE 4:**
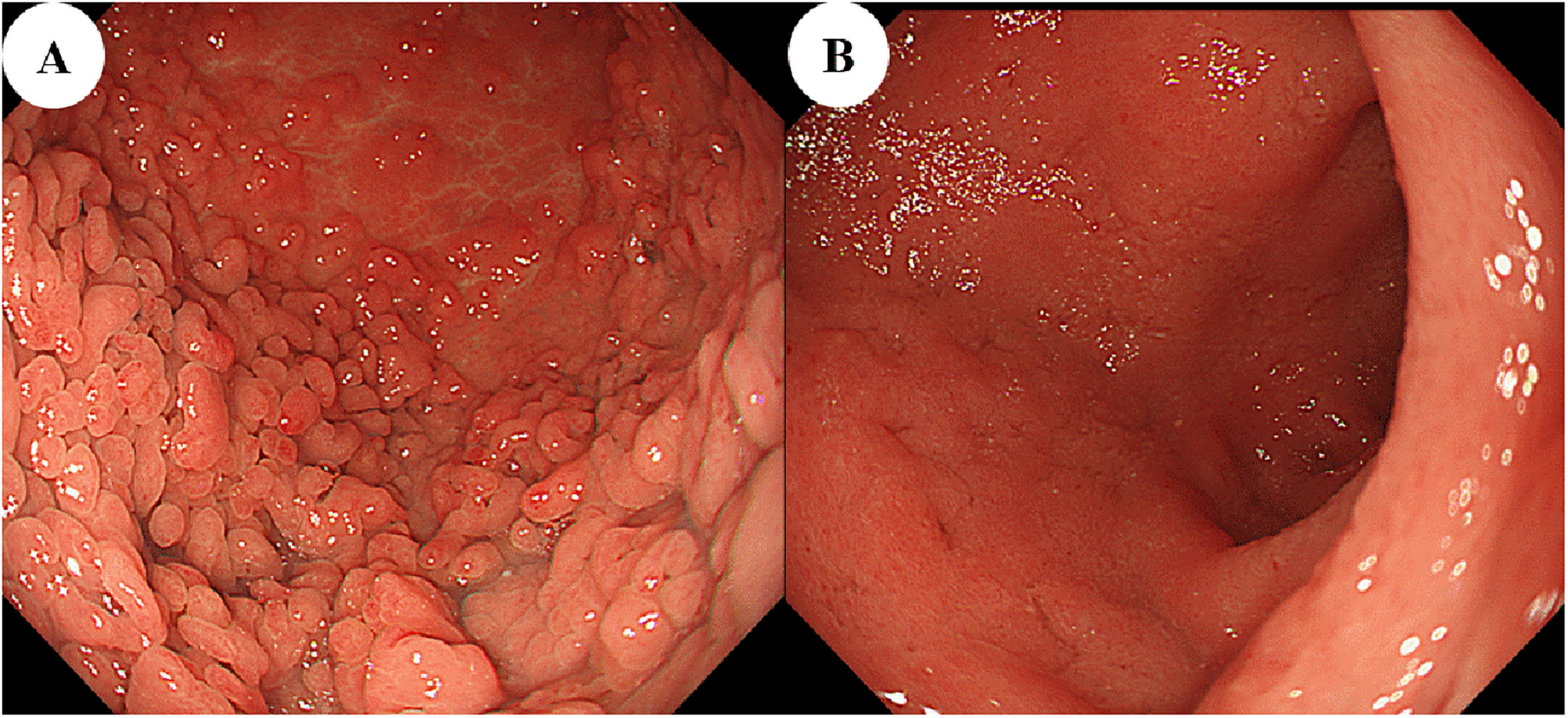
Endoscopic findings of the upper gastrointestinal tract at 6-month follow-up. **(A)** Gastric body and **(B)** duodenal bulb showing marked resolution of inflammation.

At 6 months, repeat endoscopic examinations of the UGI and lower GI tracts were performed. While an inflammatory polyp persisted in the stomach, the previously observed erythema and ulcerations had resolved (Figures 4A,B). Endoscopic remission was also confirmed in the colon. Follow-up biopsy specimens also demonstrated marked improvement in inflammatory activity. Only mild to moderate infiltration of lymphocytes and plasma cells remained in the duodenal and gastric mucosa, with no residual crypt abscesses identified.

## Discussion

Recent studies have reported upper gastrointestinal (UGI) involvement in ulcerative colitis (UC), challenging the traditional view of UC as a disease confined to the colon. Cases of diffuse duodenitis associated with UC have been documented, with endoscopic and histological findings resembling colonic UC lesions (2). In pediatric UC, UGI involvement has been described as an atypical phenotype, occurring in up to 20.5% of cases (7). Reported UGI manifestations include esophageal, gastric, and duodenal mucosal injuries, which are often nonspecific and may therefore be overlooked in clinical practice (6). Importantly, severe UGI involvement has been associated with serious complications, including intestinal perforation (5), highlighting the need for increased clinical awareness and careful assessment of the upper GI tract in selected UC patients.

In the present case, a 19-year-old male with newly diagnosed UC exhibited pronounced gastric and duodenal inflammation with spontaneous bleeding and ulcerations, confirmed both endoscopically and histologically. The upper gastrointestinal lesions closely resembled colonic ulcerative colitis in their histopathological features, including dense inflammatory cell infiltration and crypt abscesses, while granuloma formation was absent.

Differentiation from Crohn’s disease (CD) was an important diagnostic consideration, particularly given the prominent upper gastrointestinal involvement. Endoscopically, colonic inflammation was continuous without skip lesions, and no features suggestive of Crohn’s disease, such as longitudinal ulcers or a cobblestone appearance, were observed. The terminal ileum appeared normal on ileocolonoscopy. In addition, there was no evidence of small bowel involvement on computed tomography. Histologically, inflammatory changes were confined to the mucosa without features suggestive of transmural inflammation, and no granulomas were identified. Furthermore, there were no clinical features indicative of CD, such as perianal disease. Taken together, these findings support the diagnosis of UC-associated UGI involvement rather than CD or infectious gastroduodenitis, including Helicobacter pylori–associated gastritis.

Although eosinophilic infiltration was observed, the overall histological pattern, including crypt abscesses and architectural distortion, was inconsistent with eosinophilic gastrointestinal disorders. Furthermore, no histological features suggestive of drug-induced injury were identified, and infectious or malignant etiologies had been excluded based on histological evaluation.

Given the lack of established diagnostic criteria for UC-associated upper gastrointestinal involvement, we based the diagnosis on a composite assessment of multiple factors. These included (1) endoscopic findings consistent with UC, such as continuous colonic inflammation without skip lesions and no endoscopic features suggestive of Crohn’s disease, (2) histological features including crypt abscesses and absence of granulomas, and (3) exclusion of alternative diagnoses, including Crohn’s disease and infectious gastroduodenitis, based on clinical, radiological, and pathological findings. This structured approach may improve diagnostic consistency and clinical applicability in similar cases.

The lesions were accompanied by spontaneous bleeding and ulcerations and were refractory to high-dose corticosteroid therapy, indicating a severe and rapidly progressive disease course. Given the risk of further deterioration and potential complications such as perforation, escalation to urgent rescue therapy was considered necessary.

Tacrolimus, a calcineurin inhibitor that suppresses T-cell activation, has been used as an induction therapy for steroid-refractory UC, with several studies reporting rapid clinical response and mucosal healing (12). In the present case, tacrolimus was selected as induction therapy based on several clinical and contextual considerations. The patient exhibited severe upper gastrointestinal (UGI) involvement with spontaneous bleeding and ulceration that was refractory to high-dose corticosteroids, necessitating rapid and potent immunosuppression. In addition, previous studies—particularly from Japan—have suggested that tacrolimus may be comparable to infliximab, or potentially more effective in patients with severe steroid-refractory UC (13, 14). Furthermore, although cyclosporine is an established rescue therapy for acute severe UC in Western countries, it is not approved for the treatment of UC in Japan, making tacrolimus the primary calcineurin inhibitor available in this clinical setting. Because oral administration was not feasible due to persistent nausea and epigastric pain, intravenous tacrolimus was selected as rescue therapy in accordance with institutional approval. This approach resulted in rapid clinical and endoscopic improvement; however, due to tacrolimus-related adverse events, a transition to alternative maintenance therapy was required.

In the present case, tacrolimus and upadacitinib were used sequentially, which represents a mechanistically plausible therapeutic strategy for controlling both acute and sustained immune activation. Given the severe upper gastrointestinal involvement with spontaneous bleeding, prompt and potent immunosuppression was required, making tacrolimus an appropriate induction therapy.

Notably, recent network meta-analyses comparing advanced therapies have demonstrated that upadacitinib ranks among the most effective agents for achieving clinical remission and endoscopic improvement in moderate-to-severe ulcerative colitis, supporting its use in patients requiring rapid and effective disease control (15). In this clinical context, a therapy with rapid onset and broad immunomodulatory effects was considered more appropriate.

From a mechanistic perspective, tacrolimus primarily targets upstream immune activation via NFAT signaling, whereas upadacitinib may contribute to sustained disease control by suppressing downstream cytokine signaling pathways, including interleukin-6, interleukin-12/23, and interferon-mediated signaling, involved in ongoing mucosal inflammation. This sequential targeting of different levels of the immune response may have contributed to the favorable clinical course observed in this case.

However, this interpretation remains speculative, as no direct immunological analyses were performed. Further studies are required to clarify the potential role of such sequential therapeutic strategies in ulcerative colitis with upper gastrointestinal involvement.

Previous case reports have described severe gastroduodenitis associated with ulcerative colitis occurring predominantly after total colectomy, often in association with pouchitis, and successfully treated with oral or intravenous tacrolimus (9–11). In contrast, our patient developed unusually severe upper gastrointestinal involvement at the initial presentation of UC, without prior colectomy, and achieved remission through a sequential therapeutic strategy using intravenous tacrolimus. Likewise, upadacitinib, a selective Janus kinase 1 (JAK1) inhibitor with proven efficacy in moderate to severe UC (16), has not previously been reported in this clinical context. In our case, tacrolimus was effective for induction of remission but was discontinued after 3 weeks due to renal dysfunction and hypomagnesemia necessitating a planned transition to upadacitinib for maintenance therapy.

The pathogenesis of UGI involvement in UC remains incompletely understood. Sun et al. proposed that upper GI lesions may result from the homing of activated T lymphocytes—initially primed in the inflamed colon—to the upper GI mucosa via a shared mucosal immune system (6). This hypothesis supports the concept that UGI involvement represents a systemic extension of UC-related immune dysregulation rather than an independent upper GI disorder. In this context, the favorable response observed in our patient suggests that potent immunomodulatory therapies targeting T-cell–mediated pathways may be particularly effective in selected cases of UC-associated UGI involvement.

This study has several limitations. Fecal calprotectin and detailed immunological analyses, including cytokine profiling, were not assessed in this case, which may limit a more comprehensive evaluation of disease activity and underlying mechanisms. Future studies incorporating such biomarkers may help to better characterize disease activity and clarify the immunological basis of treatment response. In addition, as this is a single case report, the generalizability of these findings is limited.

To our knowledge, adult cases of UC-associated upper gastrointestinal involvement treated with upadacitinib are rare. Our case is distinctive in that severe upper gastrointestinal lesions occurred at the initial presentation of UC, without prior colectomy, and were successfully managed with a sequential therapeutic strategy using intravenous tacrolimus followed by upadacitinib. This case underscores the importance of early recognition of UGI lesions in UC and suggests a potential role for advanced immunomodulatory therapies in managing this rare but challenging manifestation of the disease.

## Data Availability

The raw data supporting the conclusions of this article will be made available by the authors, without undue reservation.
